# A novel method to demonstrate that pregnant women with polycystic ovary syndrome hyper-expose their fetus to androgens as a possible stepping stone for the developmental theory of PCOS. A pilot study

**DOI:** 10.1186/s12958-017-0282-1

**Published:** 2017-08-08

**Authors:** Roy Homburg, Anil Gudi, Amit Shah, Alison M. Layton

**Affiliations:** 1 Homerton Fertility Centre, Homerton University Hospital, E9 6SR, London, UK; 2 Harrogate & District NHS Foundation Trust, Lancaster Park Road, Harrogate, HG2 7SX UK

**Keywords:** Pcos, Developmental hypothesis, Androgens, Sebum

## Abstract

**Background:**

Polycystic ovary syndrome (PCOS), whose aetiology is unknown, is predominately a familial syndrome but confirmation of candidate genes has proved elusive. The developmental hypothesis for the origin of PCOS suggests that exposure of the fetus to excess androgens influences imprinting, leading to altered genetic expression in adult life. The aim of this pilot study was to examine whether the female fetus of a mother with PCOS is indeed exposed to excess androgens.

**Methods:**

Using sebum production in the newborn as a surrogate for exposure to excess androgens during pregnancy thisprospective case control studyexamined whether neonatal sebum excretion is greater in female infants born to PCOS mothers compared to non-PCOS. Women with known PCOS (all 3 Rotterdam criteria) (*n* = 9) and non-PCOS controls (*n* = 12), with a female fetus, were recruited at 24 weeks pregnancy and serum testosterone estimated. Sebum was measured using Sebutape® for 30 and 60 min within 24 h of birth, at 1 week, 4–6 weeks and 6 months after birth in both mother and child. Sebum excretion was measured in mother and child in the same site at each time frame and consistently. All semi-quantitative sebum excretion estimations were compared (t-test) between the two groups and correlated with testosterone concentrations during pregnancy.

**Results:**

**I**n this pilot study, 21 women completed the 6 month examination period (PCOS group (*n* = 9) and controls (*n* = 12). Mean testosterone was 6.2 nmol/L (normal <3.1 nmol/L) in PCOS mothers and 2.75 nmol/L in controls at 24 weeks pregnancy. At all time frames, the results of sebum excretion at 30 and 60 min were consistent. The sebum excretion of mothers in both groups was fairly constant from birth throughout 6 months. All babies were born between 37 and 41 weeks gestational age. Six of nine newborns had detectable sebum excretion at birth in the PCOS mothers group compared to 1 of 12 in the controls (*P* = 0.01).

**Conclusions:**

These results suggest that women with PCOS could hyper-expose their fetus to androgens in-utero and that this may be detected using a simple novel test within 24 h of birth to predict development of PCOS in adult life and induce research to eliminate its symptoms.

**Trial registration:**

NCT 02654548.Clinical Trials UK.Retrospectively registered 11/1/16.

## Background

Polycystic ovary syndrome (PCOS) is the commonest endocrinopathy in women andthe commonest cause of anovulatory infertility. The aetiology is largely unknown. It has a high degree of heritability but the search for confirmed candidate genes has proved elusive. The developmental hypothesis for the origin of PCOS, based on the Barker hypothesis [1], suggests that exposure of the fetus to excess androgens influences imprinting, leading to altered genetic expression in adult life. This re-programming of the female reproductive axis is thought to induce the features of PCOS in later life: oligo/anovulation, polycystic ovaries, hyperandrogenism and insulin resistance. Additionally, the woman with PCOS has a more than two-fold risk of developing the metabolic syndrome, type-2 diabetes and cardiovascular disease. The extent of the human suffering and economicburden of PCOS must encourage research into its origins which would surely reveal the possibility of innovative treatments and possible preventative measures for this very prevalent condition.

Studies on pregnant monkeys injected with testosterone [2], prevalenceof tom-boy behaviour of female children [3] and even an increased incidence of autism spectrum disorders [4], have all corroborated the possible influence of hyper-exposure to testosterone in-utero. Prenatal exposure of experimental animals to androgens produces endocrine and metabolic alterations in offspring resembling those in PCOS [5–8]. These latest studies clearly suggest thatfetal over-exposure to androgens could be the main factor in PCOS pathogenesis.

The first step in proving the developmental hypothesis for the origin of PCOS is evidence that the susceptible fetus may indeed be exposed to a hyperandrogenic environment. Androgens influence the production of sebum by exerting an effect on sebaceous gland activity [9]. At the 18th week of fetal life, the sebaceous glands are fully differentiated and at birth they are fully functional. If the fetus is exposed to maternal androgens in utero, neonatal seborrhoea should provide a surrogate measure of the extent of in-utero exposure of the fetus to androgensas this would translate to increased sebaceous gland activity in the neonate.

The aim of this pilot study was to examine whether the female fetus of a mother with PCOS is indeed exposed to excess androgens.

## Methods

This is a prospective case control study to discern whether neonatal sebum excretion is greater in female infants born to PCOS mothers compared to non-PCOS.Followinglocal ethical committee permission (Central London, 13/LO/0510)and signing of informed consent, women with known PCOS (all three Rotterdam criteria [10])(*n* = 9) and non-PCOS controls (*n* = 12), with a female fetus, were recruited at 24 weeks pregnancy and serum testosterone estimated. They were next seen in labour at a single University Hospital.

**Fig. 1:**
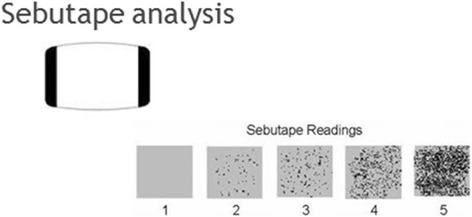
The absorbent paper technique of measuring sebum excretion rates (Sebutape®), on a semi-quantitative scale from 1 (no sebum) to 5 (serious seborrhoea)

The absorbent paper technique (Sebutape®),is a rectangular white adhesive tape containing micro-cavities to trap sebum. The surface is hydrophobic. As the sebum issues from the follicles it is trapped in the cavities and the tape becomes transparent. Sebum excretion can therefore be assessed and the number of active secreting sebaceous follicles over an 8x8mm area captured. The skin is pre-cleaned with an alcohol wipe to remove causal sebum and the contact time, pressure, temperature and humidity are standardised to give reliable, reproducible results (Fig. 1). Once the process is completed the sebutapes are placed onto a black card where the transparent areas are seen as black dots.

Sebum excretion was measured for 30 min and 60 min to ensure slow secreting follicles were captured. This was done within 24 h of birth, at 1 week, 4–6 weeks and 6 months after birth in both mother and child. Sebum excretion was measured at the same site at each time frame as the amount of sebum per unit skin surface is generally constant in an individal and on a given anatomical site. This was done consistently for mother and baby.

All semi-quantitative sebum excretion estimations were compared (t-test) between the two groups and correlated with testosterone concentrations during pregnancy.

## Results

As would be expected, women with PCOS had a significantly higher mean BMI and a higher mean serum total testosterone concentration at 24 weeks gestation compared with controls but the groups were otherwise demographically similar (Table 1). Six of the 9 in the PCOS group compared with 2 of the 12 controls had a serum testosterone >3.12 pmol/L.

**Table 1:** Details of PCOS mothers and controls

	PCOS (*n* = 9)	Controls (*n* = 12)	*P* value
Mean age (SD)	30.1 (4.76)	31.6 (3.5)	0.42
Mean BMI (SD)	31.1 (4.7)	25.2 (4.1)	0.007
Mean T (nmol/l)(SD)	6.2 (5.6)	2.75 (1.4)	0.11

*BMI* body mass index, *T* total serum testosterone, *SD* standard deviation, *NS* not significant

Results of sebum excretion measured at 30 min and 60 min were almost identical.

Maternal sebum production was reasonably stable throughout from 24 weeks pregnancy to 6 months post natal. Any detectable neonatal sebum within 24 h of delivery was no longer evident 4 weeks later.

Serum testosterone in mothers with sebum positive newborn was 7.03 +/− 6.3 pmol/l compared with 2.77 +/− 1.4 in the remainder (*P* = 0.04)(Table 2). Neonatal sebum excretion, which couldindicate in-utero exposure to excess androgens, was evident in 6 of 9 infants born to mothers with PCOS compared to 1of 12 mothers without PCOS (*P* = 0.005). There was no difference in the gestational age at birth between the sebum positive and sebum negative newborn (38.4 vs 38.8 weeks respectively) nor in the mode of delivery although sebum positive newborn had a significantly higher birth weight (Table 2).

**Table 2:** Comparison of sebum positive and sebum negative groups within 24 h of birth

	Sebum positive (*n* = 7)	Sebum negative (*n* = 14)	*P*
PCOS group	6	3	
Controls	1	11	0.005
Mean birth weight (kg)(SD)	3.58 (0.44)	3.1 (0.41	0.02
Mean gestational age (weeks)	38.4	38.8	NS
Mode of delivery			
Spontaneous	3	6	
Vacuum extraction	0	2	
Caesarean section	4	6	NS
Mothers			
Mean T (nmol/l)(SD)	7.03 (6.3)	2.77 (1.4)	0.04
BMI (SD)	31.7 (4.84)	25.7 (4.19)	0.02

*BMI* body mass index, *T* serum testosterone, *SD* standard deviation, *NS* not significant

## Discussion

Although animal studies laid the foundations of the developmental hypothesis for PCOS etiology, in humans this theory was doubtedfor many years. Although maternal testosterone concentrations are significantly higher in both serum and amniotic fluid compared to normal controls [11–13],the human fetus is protected from the effects of excessive maternal androgens by a combination of high concentrations of plasma binding proteins and a high level of placental aromatase activity. In human pregnancy, androgens are metabolized to estrogens by placental P450 aromatase [14].

The hormonal alterations during pregnancy in PCOS women show that the uterine environment in PCOS is most probably hyper-androgenic.The results of initial autopsies of placentae from women with PCOS, showing macroscopic and microscopic alterations compared to healthy controls [15] were also confirmed with altered histopathological findings in PCOS placentae in recent studies [8].

When placental tissues from women with PCOS and healthy women were compared, changes in the activities of important enzymes for steroid synthesis were noticed. Specifically higher 3β-HSD-1 activity and lower P450 aromatase activity were seen in placenta tissue of women with PCOS. These alterations could increase androgen production during pregnancy according to the authors, as these enzymes are principally linked to placental steroidogenesis [8]. It seems that placental altered steroidogenesis could play a key role in PCOS pathogenesis.

This pilot study has shown that a simple method for detecting fetal exposure to excess androgens is feasible. Clearly 30 min measuring was sufficient and the fact that sebum excretion was unmeasurable in the newborn one month after birth strongly suggests that the origin of the androgen hyper-exposure in PCOS is maternal and not fetal and that any effect of this androgen exposure originates during pregnancy rather than afterwards. Previous studies employing different methods of measuring sebum excretion, one very small [16] and one that also included males and had no mention of PCOS [17] found high perinatal sebum excretion in both sexes.

Following the results of this pilot study we are now proceeding with the use of more robust methods for sebum excretion measurement in order to confirm our original findings suggesting that women with PCOS hyper-expose their fetus to androgens.

## Conclusions

The higher sebum production in babies of PCOS mothers strongly suggests hyper-exposure of the fetus in-utero to androgens. Confirmation is required but in line with the developmental theory of PCOS, this simple novel method could potentially be used within 24 h of birth to predict development of PCOS in adult life and induce research to eliminate the symptoms of PCOS.

## References

[1] Barker DJ (1990). The fetal and infant origins of adult disease. Br Med J.

[2] Abbott DH, Barnett DK, Bruns CM, Dumesic DA (2005). Androgen excess fetal programming of female reproduction: a developmental aetiology for polycystic ovary syndrome?. Hum Reprod Update.

[3] Hines M, Golombok S, Rust J, Johnston KJ, Golding J (2002). Testosterone during pregnancy and gender role behavior of pre-school children: a longitudinal, population study. Child Dev.

[4] Kosidou K, Dalman C, Widman L, Arver S, Lee BK, Magnusson C, Gardner RM (2016). Maternal polycystic ovary syndrome and the risk of autism spectrum disorders in the offspring: a population-based nationwide study in Sweden. Mol Psychiatry.

[5] Abbott DH, Zhou R, Bird IM, Dumesic DA, Conley AJ (2008). Fetal programming of adrenal androgen excess: lessons from a nonhuman primate model of polycystic ovary syndrome. Endocr Dev.

[6] WuXY LZL, WuCY LYM, Lin H, Wang SH, Xiao WF (2010). Endocrine traits of polycystic ovary syndrome in prenatally androgenized female Sprague-Dawley rats. Endocr J.

[7] Padmanabhan V, Veiga-Lopez A (2013). Sheep models of polycystic ovary syndrome phenotype. Mol Cell Endocrinol.

[8] Maliqueo M, Lara HE, Sánchez F, Echiburú B, Crisosto N, Sir-Petermann T (2013). Placental steroidogenesis in pregnant women with polycystic ovary syndrome. Eur J Obstet Gynecol Reprod Biol.

[9] Zouboulis CC (2004). Acne and sebaceous gland function. Clin Dermatol.

[10] Rotterdam ESHRE/ASRM-Sponsored PCOS Consensus Workshop Group (2004). Revised 2003 consensus on diagnostic criteria and long-term health risks related to polycystic ovary syndrome (PCOS). Hum Reprod.

[11] Sir-Petermann T, Maliqueo M, Angel B, Lara HE, Perez-Bravo F, Recabarren SE (2002). Maternal serum androgens in pregnant women with polycystic ovarian syndrome: possible implications in prenatal androgenization. Hum Reprod.

[12] Caanen MR, Kuijper EA, Hompes PG, Kushnir MM, Rockwood AL, Meikle WA, Homburg R, Lambalk CB (2016). Mass spectrometry methods measured androgen and estrogen concentrations during pregnancy and in newborns of mothers with polycystic ovary syndrome. Eur J Endocrinol.

[13] Palomba S, Marotta R, Di Cello A, Russo T, Falbo A, Orio F, Tolino A, Zullo F, Esposito R, La Sala GB (2012). Pervasive developmental disorders in children of hyperandrogenic women with polycystic ovary syndrome: a longitudinal case-control study. Clin Endocrinol.

[14] Thompson EA, Siiteri PK (1974). The involvement of human placental microsomal cytochrome P-450 in aromatization. J Biol Chem.

[15] Palomba S, Russo T, Falbo A, Di Cello A, Tolino A, Tucci L, La Sala GB, Zullo F (2013). Macroscopic and microscopic findings of the placenta in women with polycystic ovary syndrome. Hum Reprod.

[16] Henderson CA, Taylor J, Cunliffe WJ (2000). Sebum excretion rates in mothers and neonates. Br J Dermatol.

[17] Agache P, Blanc D, Barrand C, Laurent R (1980). Sebum levels during the first year of life. BrJ Dermatol.

